# Recent Developments and Biological Activities of *N*-Substituted Carbazole Derivatives: A Review

**DOI:** 10.3390/molecules200813496

**Published:** 2015-07-23

**Authors:** Maryam Bashir, Afifa Bano, Abdul Subhan Ijaz, Bashir Ahmad Chaudhary

**Affiliations:** Department of Pharmacy, Bahauddin Zakariya University, Multan 60800, Pakistan; E-Mails: marach13@hotmail.com (M.B.); afifa28mc@gmail.com (A.B.); abdulsubhan@bzu.edu.pk (A.S.I.)

**Keywords:** *N*-substituted carbazoles, antimicrobial, anticancer, neuroprotective

## Abstract

Carbazoles represent an important class of heterocycles. These have been reported to exhibit diverse biological activities such as antimicrobial, antitumor, antiepileptic, antihistaminic, antioxidative, anti-inflammatory, antidiarrhoeal, analgesic, neuroprotective and pancreatic lipase inhibition properties. A series of carbazole derivatives such as *N*-substituted carbazoles, benzocarbazoles, furocarbazoles, pyrrolocarbazoles, indolocarbazoles, imidazocarbazoles, *etc.* have been synthesized. The *N*-substituted derivatives have gained the attention of researchers due to their therapeutic potential against neurological disorders and cell proliferation. Herein an attempt is made to review the medicinal importance of recently synthesized *N*-substituted carbazoles.

## 1. Introduction

Heterocycles are inextricably woven into the life processes [1]. The importance of heterocycles in drug discovery is one of the major areas in medicinal chemistry [2]. There are a vast number of pharmacologically active heterocyclic compounds, many of which are being used clinically, such as vincristine, morphine, chloroquine, meperidine, sulphadiazine, *etc.* Sulphur- and nitrogen- containing heterocyclic compounds have maintained the interest of researchers through decades of historical development of organic synthesis [3,4].

**Figure 1:**
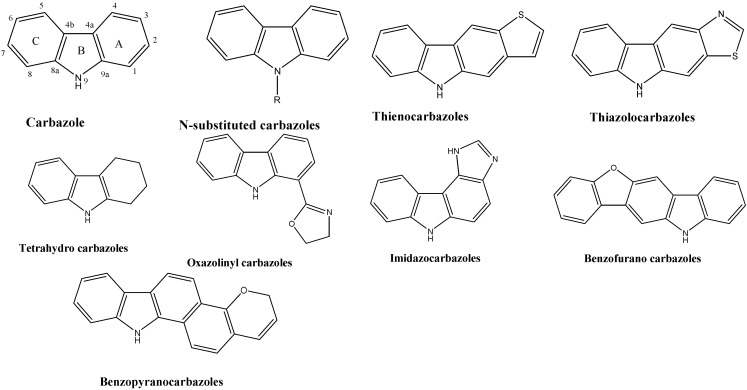
Structures of various classes of carbazoles.

Carbazole is an aromatic heterocyclic organic compound. It has a tricyclic structure, consisting of two six membered benzene ring fused on either side with a five membered nitrogen-containing ring. Carbazole and its derivatives are an important type of nitrogen containing heterocyclic compounds that are widespread in nature [5]. Various classes of carbazoles are given in Figure 1. The Carbazole ring is present in a variety of naturally occurring medicinally active substances [6] e.g., carbazomycins [7,8] and murrayafoline A [9]. Series of carbazole derivatives including oxazinocarbazoles, isoxazolocarbazolequinone, pyrido-carbazolequinone [10], tetrahydrocarbazoles [11], benzocarbazoles [12], furo-carbazoles [13], pyridocarbazoles [14], pyrrolo-carbazoles [15,16], indolocarbazoles [17], oxazolinyl carbazoles [18], thienocarbazoles [19], imidazocarbazoles [20], thiazolocarbazoles [21], benzopyrano-carbazoles [22], benzofurano-carbazoles [23] and *N*-substituted carbazoles have been synthesized and are well known for their pharmacological activities [24] such as antioxidant [25], anti-inflammatory [26], antibacterial [27], antitumor [28,29], anticonvulsant [30], antipsychotic [31], antidiabetic [32], larvicidal [33] properties, *etc.* Keeping in view the so vast therapeutical potential of carbazoles, this review will summarize the biological activities so far reported for the *N*-substituted carbazoles.

## 2. Biological Activities of *N*-Substituted Carbazoles

### 2.1. Antimicrobial Activity

The Spread of drug resistant bacteria has badly affected the efficiency of many known antibacterial agents [34], while the emergence of fungal infections in the immuno-compromised population has also significantly increased over past few decades [35,36]. Carbazoles are considered to be one of the important classes of antimicrobial agents [37,38].

**Figure 2:**
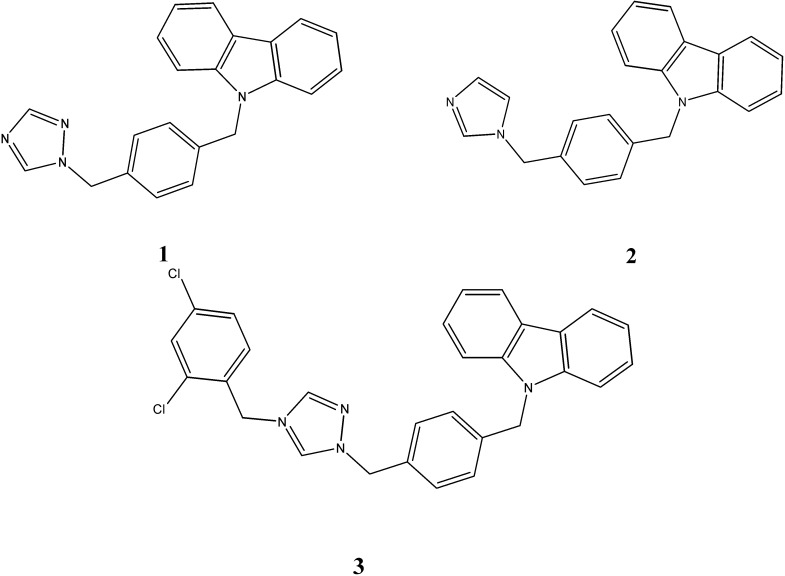
Structures of imidazole and triazole carbazoles **1**–**3**.

Zhang *et al.* [39] reported the antibacterial and antifungal activities of series of *N*-substituted carbazoles. It has been observed that introduction of 1,2,4-triazole moiety in carbazoles (compound **1**) resulted in an increase of antifungal activity against *C. albicans,* with a minimum inhibitory concentration (MIC) of 2–4 µg/mL. The introduction of an imidazole moiety (compound **2**) seems to be favourable for antibacterial efficacy against *S. aureus*, *B. subtilis*, *E. coli*, methicillin resistant *S. aureus* (*MRSA*), *P. aeruginosa* and *B. proteus* (MIC 1–8 µg/mL). The carbazole triazolium compound, a quaternization product of triazole **3**, displayed excellent antibacterial and antifungal activities against all strains with MIC values ranging from 1.0 to 64 µg/mL (Figure 2).

A series of some novel *N*-substituted derivatives of 2,3,4,4a,9,13c-hexahydro-7-isopropyl-1,4a-dimethyl-1*H*-dibenzo[*a*,*c*]carbazole-1-carboxylic acid methyl esters were synthesized by Gu *et al.* [40]. These newly synthesized compounds have been evaluated for their antimicrobial activity. The *N*-ethyl-[*N*-methyl-piperazinyl] derivative **4** showed antibacterial activity against *B. subtilis*, *S. aureus*, *E. coli*, *P. fluorescens* and antifungal activity against *C. albicans*, *A. niger* with MIC values ranging from 1.9 to 7.8 µg/mL. The *N*-ethyl-[2-methyl-5-nitro imidazole] derivative **5** exhibited antimicrobial activity against *B. subtilis* (MIC 0.9 µg/mL) comparable to that of the reference drug (amikacin).

**Figure 3:**
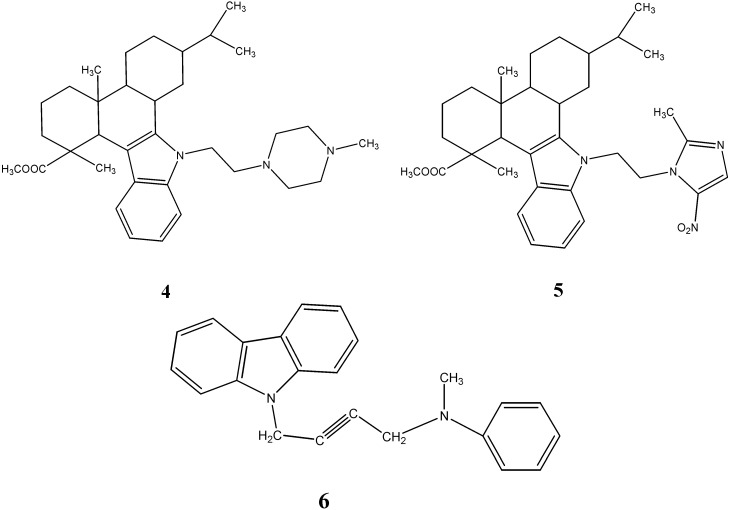
Structures of acetylenic amine and dibenzo-carbazoles **4**–**6**.

Kaissy *et al.* [41] introduced an efficient procedure for the synthesis of *N*-acetylenic aminocarbazole derivatives. These have been subjected to bioassay against *B. subtilis*, *S. aureus*, *E. coli* and *P. aeruginosa*. The compound *N*-[1-buto-2y-nyl-4(*N*ʹ*N*ʹ-methyl-phenyl]carbazole (**6**) exhibited very highly specific activity against *E. coli*, a Gram negative bacterium, with a zone of inhibition of 25 mm in diameter at a concentration of 800 µg/mL (Figure 3).

**Figure 4:**
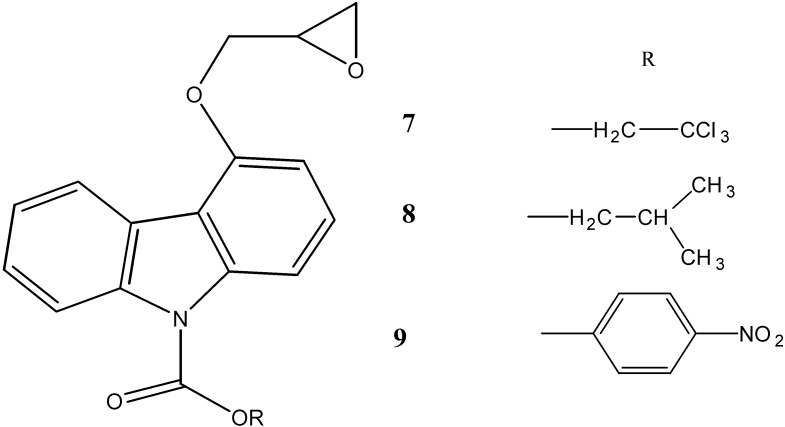

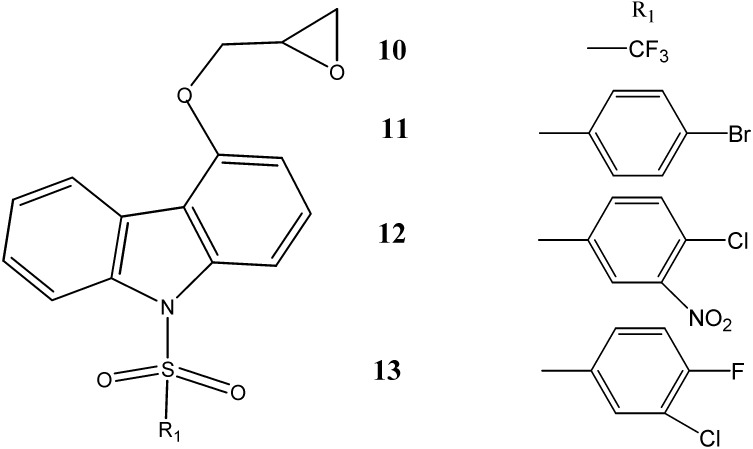
Structures of carbamate carbazoles **7**–**9** and sulphonamide carbazoles **11**–**13**.

*N*-substituted carbamates [(substituted phenyl/aliphatic-4-oxiran-2yl-methoxy)-9*H*-carbazole-9-carboxylate] and sulphonamides [9-(substituted phenyl-sulphonyl)-4-(oxiran-2yl-methoxy)-9*H*-carbazole] **7**–**13** have been synthesized by Reddy *et al.* [42] and evaluated for their antimicrobial activities. The compounds **7**, **9**, **10** and **13** showed excellent antibacterial activities against *S. aureus*, *B. subtilis*, *E. coli* and antifungal activities against *A. niger*, *C. albicans* and *F. oxysporium*. The zones of inhibition were in the range of 12.6–22.3 mm in diameter at a concentration of 100 µg/mL (Figure 4).

**Figure 5:**
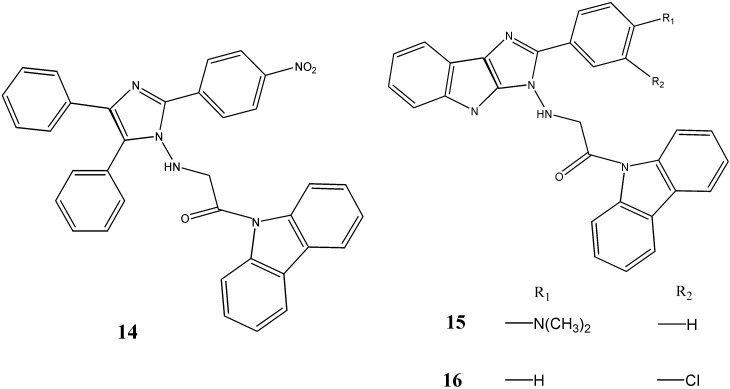
Structures of hydrazinoacetyl carbazoles **14**–**16**.

Kumar *et al.* [43] reported the synthesis of ^9^*N*-(hydrazinoacetyl)-carbazoles which have been evaluated for the potential antimicrobial activity. The carbazole derivatives containing imidazole and indole-imidazole moieties such as 1-carbazole-9-yl-2-(4-nitro-phenyl)-4,5-diphenyl-1*H*-1-yl-amino)-ethanone (**14**) and 1-carbazole-9-yl-2-(substituted phenyl)-1,4-dihydroimidazo[4,5-*b*] indol-1-yl-amino)-ethanones **15** and **16** were found to be the most potent against *B. subtilis*, *S. aureus*, *E. coli* and *K. pneumoniae* with zones of inhibition of 10.3–15.4 mm in diameter at MIC values ranging from 6.2 to 50 µg/mL (Figure 5).

**Figure 6:**
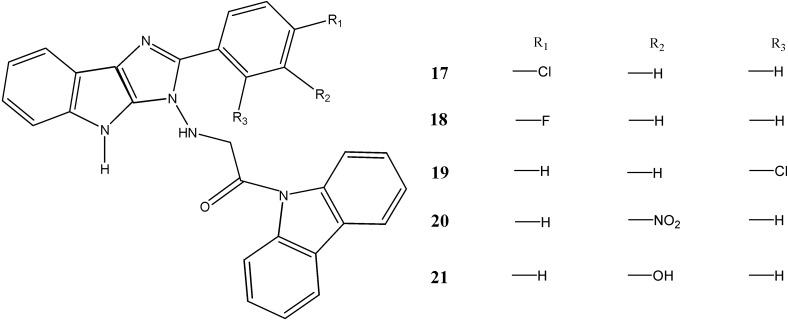
Structures of imidazo-indole carbazoles **17**–**19**.

The 1-carbazole-9-yl-2-(substituted phenyl)-1,4-dihydroimidazo-[4,5]-indole-1-yl-amino-ethanones **17**–**21** synthesized by Kaushik *et al.* [44] were subjected to bioassays for antibacterial activity against *S. aureus*, *B. subtilis*, *P. aeruginosa*, *E. coli* and antifungal activity against *C. albicans*, *A. niger* by the disc diffusion method. The compounds **18** and **20** showed potent antibacterial activity against all bacterial strains, with zones of inhibition of 16.82–26.08 mm in diameter at a concentration of 50 µg/mL, whereas the compounds **17**–**21** showed significant antifungal activity against *C. albicans* and *A. niger* with zones of inhibition of 7.91–16.8 mm in diameter at a concentration of 50 µg/mL (Figure 6).

**Figure 7:**
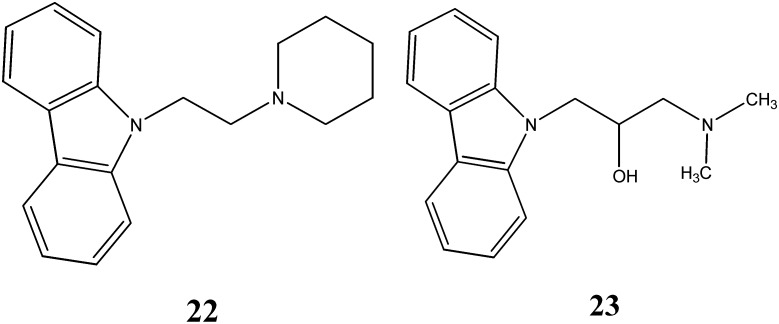
Structures of piperidinyl carbazole **22** and wiskostatin **23**.

Segall and coworkers [45] reported a potent antifungal activity of *N*-alkylated carbazoles namely 6,11-dihydro-2-methoxy-11-[2-(1-piperidinyl)]ethyl-5*H*-benzo[*a*]carbazole (**22**) and wiskostatin (**23**) against *C. albicans* with MIC < 11 µM and 100 µM, respectively (Figure 7). Wiskostatin, an inhibitor of neuronal Wiskott-Aldrich syndrome protein (N-WASP)-mediated actin polymerization *in vitro* in relation to WASP, was identified as antifungal agent [46].

**Figure 8:**
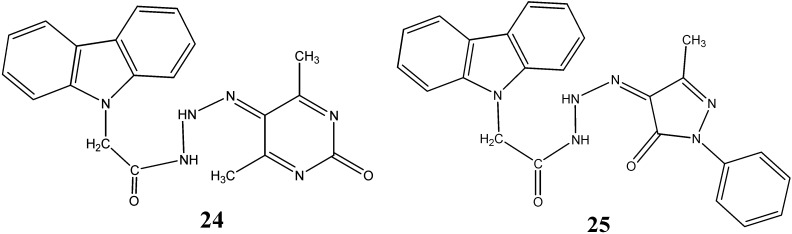
Structures of pyrimidine carbazole **24** and pyrazole carbazole **25**.

Two *N*-substituted carbazoles, 5-[3-(9*H*-carbazol-9-ylacetyl)triazanylidene]-4,6-dimethylpyrimidin-2(5*H*)-one (**24**) and 4-[3-(9*H*-carbazol-9-yl acetyl)-triazanylidene]-5-methyl-2-phenyl-2,4-dihydro-3*H*-pyrazol-3-one (**25**) have been synthesized by Salih *et al.* [47] (Figure 8). 

They showed promising antifungal activity against *C. albicans* and *A. fumigatus* with MIC values ranging from 8.7 to 10.8 µg/mL and antibacterial activity against *S. aureus*, *B. subtilis*, *E. coli* with MIC values ranging from 1.1 to 10.3 µg/mL. The lipophilic character of these compounds may facilitate the crossing through biological membranes of microorganism and thereby inhibit their growth.

**Figure 9:**
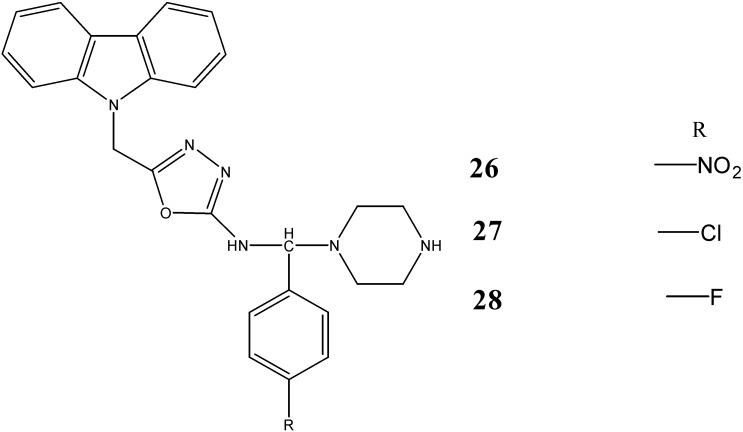
Structures of piperazinyl-oxadiazole carbazoles **26**–**28**.

A series of *N*-substituted-carbazoles, synthesized by *Sharma et al.* [48], have been screened for their antimicrobial activities. The compounds 5-[(9*H*-carbazol-9-yl)methyl]-*N*-[(substituted phenyl)(piperazin-1-yl)methyl]-1,3,4-oxadiazol-2-amines **26**, **27** and **28** have displayed antimicrobial activity against bacterial strains (*S. aureus*, *B. subtilis*, *E. coli*, *P. aeruginosa*) and fungal strains (*C. albicans* and *A. niger*) with zones of inhibition of 11.1–24.0 mm in diameter at a concentration of 50 µg/mL (Figure 9).

### 2.2. Anti-Cancer Activity

Cancer has emerged as one of the most alarming disease in the last few decades throughout the world. It is a multifactorial disease contributing towards uncontrolled growth and invasion of abnormal cells leading to the formation of tumors [49]. Pim-kinases control various proteins involved in significant biological processes such as cell cycle progression and apoptosis. Over expression of pim-kinases have been observed in human leukaemia and lymphoma, prostate, pancreatic and colon cancer contributed to tumorigenesis. For these reasons, pim-kinases are considered as important targets for the development of new anticancer drugs.

**Figure 10:**
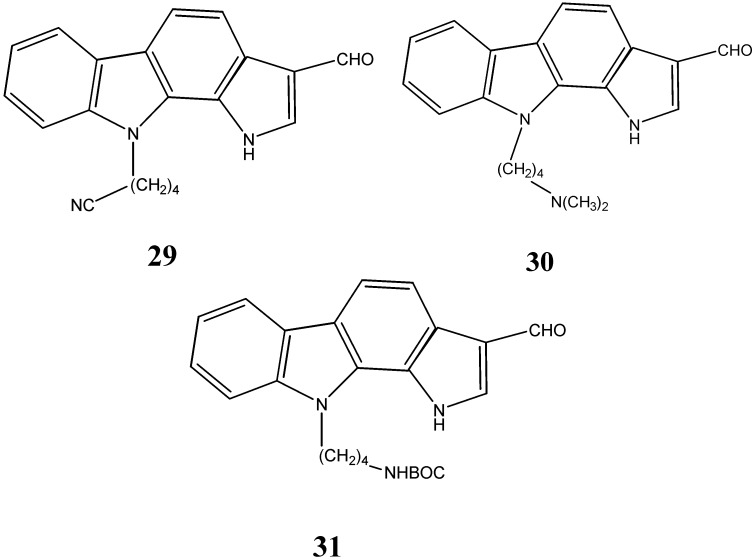
Structures of pyrrolo-carbazoles **29**–**31**.

A series of *N*-substituted carbazoles synthesized by *Akue-Gedu et al.* [16] have been studied for their antiproliferative activity. These *N*-substituted pyrrolocarbazoles **29**, **30** and **31** were found to be most potent inhibitors for pim-kinase activity with IC_50_ in the nanomolar range (46–75 nM) and have demonstrated antiproliferative activities against three human cancer cell lines, PA1 (ovarian carcinoma) PC3 and DU145 (prostatic carcinoma) with MIC values in the range of 8–20 µM (Figure 10).

**Figure 11:**
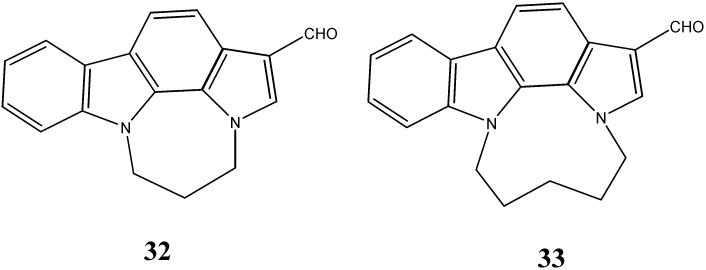
Structures of *N*_1_-*N*_10_ bridged pyrrolo-carbazoles **32** and **33**.

Giraud *et al.* [50] have synthesized *N*_1_-*N*_10_-bridged pyrrolo [2,3-a] carbazoles. The ability of these compounds to inhibit pim-kinases has been evaluated. The compounds 5,6-dihydro-4*H*-indolo[1,2,3-*ef*] pyrrolo[3,2,1-*jk*][1,5] benzodiazepine-1-carbaldehyde (**32**) and 5,6,7,8-tetrahydro-4*H*-indolo[1,2,3-*gh*] pyrrolo[3,2,1-*lm*][1,5] benzodiazonine-1-carbaldehyde (**33**) showed potent activity with nanomolar inhibitory potencies against the acute myeloid leukemia IPC-81 cell line, which is a good predictor of leukaemia therapy (Figure 11).

**Figure 12:**
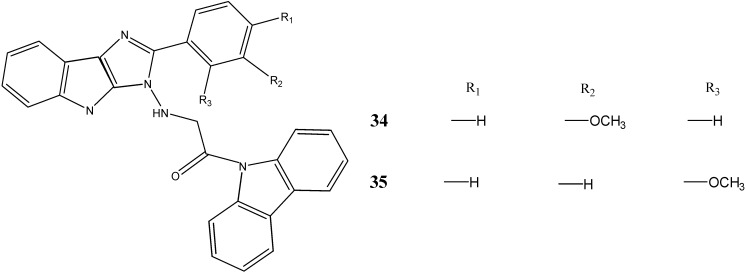
Structures of hydrazinoacetyl carbazoles **34** and **35**.

The 1-carbazole-9-yl-2-(substituted phenyl)-1,4-dihydroimidazo [4,5] indole-1-yl-amino-ethanones synthesized by Kumar *et al.* [43] have been evaluated for antitumor potential for laryngeal carcinoma cell lines (HEP_2_) and Ehrlich’s Ascites Carcinoma (EAC) cells. The compounds **15**, **34** and **35** were found to be active against tumor cell lines (Figure 12). The activity may be attributed due to the presence of electron donating group which may increase the basicity of the compound.

**Figure 13:**
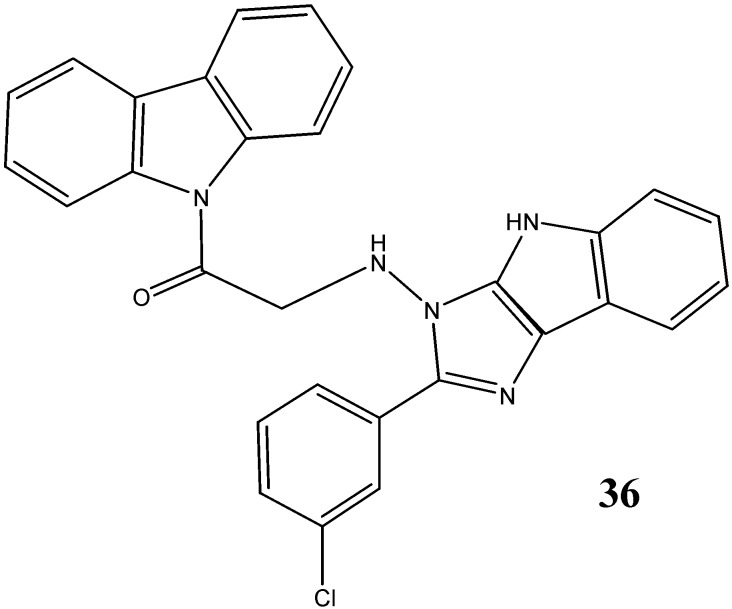
Structure of imidazo-indole carbazole **36**.

A study was carried out by Kaushik *et al.* [44] on A549 cell lines in order to explore the anti-tumor potential of *N*-substituted carbazoles and the compounds **17**, **18**, **19** and **36** were found to be active (Figure 13). A fluoro group at *para*-position in compound **18** makes its anticancer activity more significant.

**Figure 14:**
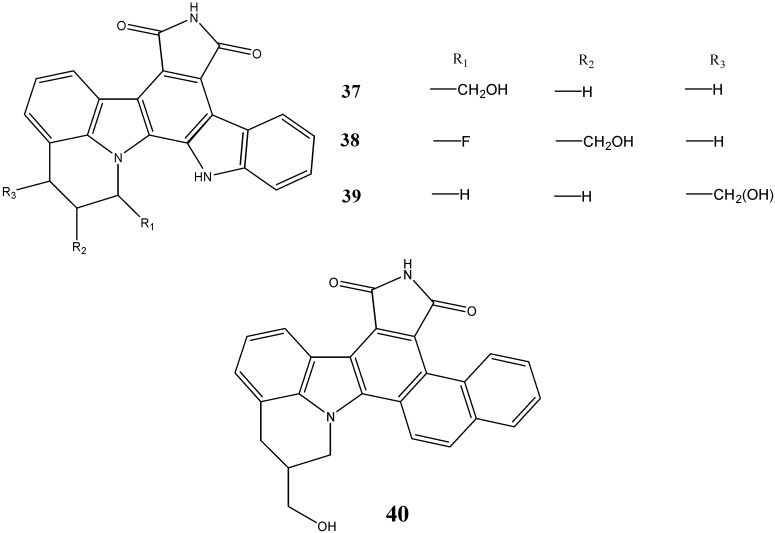
Structures of indolo- and naphtho-carbazoles **37**–**40**.

Zhu *et al.* [51] have documented the synthesis of *N*-annulated indolocarbazoles and further evaluated their cyclin dependent kinases (CDKs) inhibitory activity. The compounds **37**, **38** and **39** were found to be potent inhibitors of cyclin D1/CDK4 and were effective antiproliferative agents against two human carcinoma cell lines, HCT116 (colon) and NCI-460 (lung). The replacement of the indole moiety with a naphthyl group (compound **40**) also resulted in potent antiproliferative activity, with MIC values ranging between 0.37 and 0.96 µM (Figure 14).

**Figure 15:**
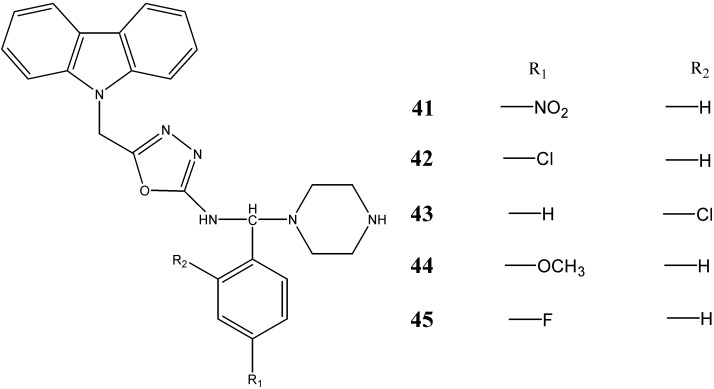
Structures of piperazinyl-oxadiazole carbazoles (**41**–**45**).

*N*-substituted carbazoles, synthesized by Sharma *et al.* [48], have been screened for their antitumor activity. The compounds 5-[(9*H*-carbazol-9-yl)-methyl]-*N*-[(substituted phenyl)(piperazin-1-yl)methyl]-1,3,4-oxadiazol-2-amines **41**–**45** were found to be effective against human breast cancer cell lines (MCF-7). The LC_50_ values (µg/mL) of compounds **41**-**45** were 60.6, 60.2, 53.6, 80.0 and 35.6, respectively (Figure 15).

**Figure 16:**
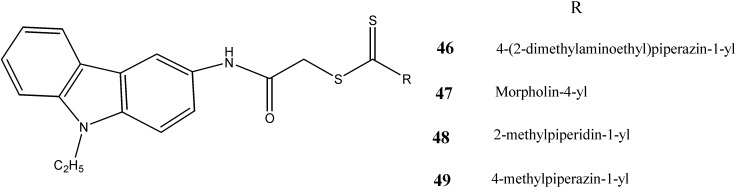
Structures of *N*-ethyl carbazoles **46**–**49**.

Ciftci *et al.* [52] have studied the apoptotic effects of *N*-ethyl-carbazole derivatives on A549 lung carcinoma and C6 glioma cell lines. The compounds **46** (IC_50_ = 5.9 µg/mL) and **47** (IC_50_ = 25.7 µg/mL) were found to be highly active against C6 and A549 cancer cell lines, respectively whereas compounds **48** and **49** showed moderate cytotoxic activity against both cell lines (Figure 16).

**Figure 17:**
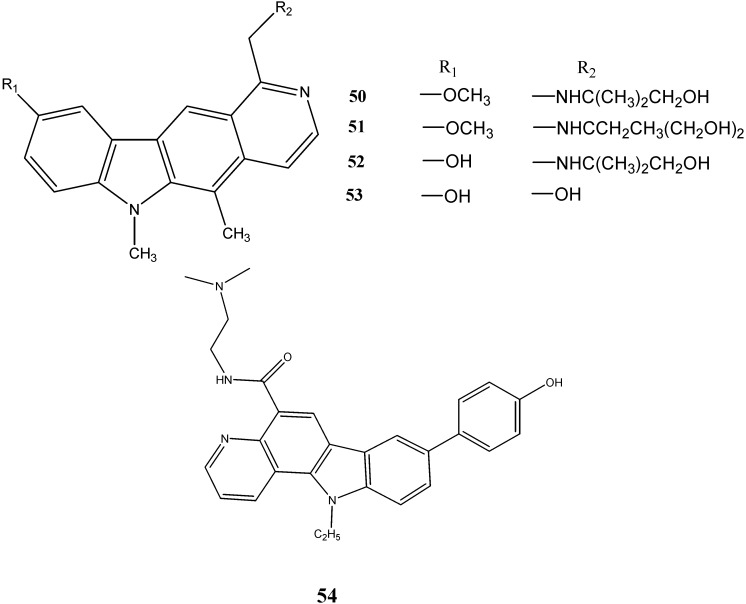
Structures of pyrido-carbazoles **50**–**54**.

Howorko *et al.* [53] have reported the cytotoxic potential of compounds **50**–**53** against three human tumor cell lines, namely CCRF/CEM (T lymphoblast leukemia), A549, and MCF7 cell line. In another study, carried out by Li *et al.* [54], the compound **54** was found to be cytotoxic against cell line A549 (IC_50_ = 0.07 µM) and colon cancer HT29 cells (IC_50_ = 0.11 µM). The structures of these compounds are given in Figure 17.

Roy *et al.* [55] have synthesized two indolocarbazoles and reported that compound **55** was found to be a novel check point kinase (ChK1) inhibitor.

Nakamura *et al.* [56] have studied the antitumor activity of a novel *N*-substituted carbazole, namely {[12,13-dihydro-5-[2-(dimethylamino)-ethyl]-4*H*-benzo[*c*]pyrimido[5,6,1-*jk*]carbazole-4,6,10-(5*H*,-11*H*)-trione hydrochloride]} (**56**). It inhibited topoisomerase II activity at a concentration of 2.5 µM which is a 10 times lower concentration than that of etoposide (standard).

**Figure 18:**
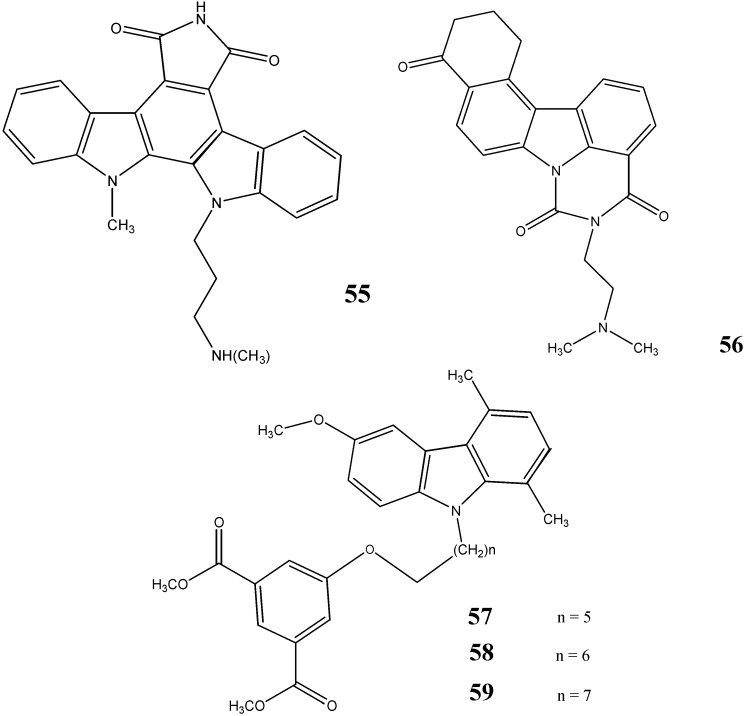
Structures of indolo-, pyrimido- and *N*-alkyl-carbazoles **55**–**59**.

Signal transducers and activators of transcription (STATs) are latent cytoplasmic transcription factors that upon activation, results into the transduction of signals from the cell membrane to the nucleus. The seven STATs namely STAT1, STAT2, STAT3, STAT4, STAT5A, STAT5B and STAT6 have so far been identified in mammals. Among them, STAT3 is the most intimately linked to tumorigenesis. Saturnino *et al.* [57] have synthesized a series of *N*-alkylcarbazole derivatives and evaluated their activity on STAT3. Compounds **57**–**59** were revealed to inhibit the STAT3 activation by 50%, 90% and 95%, respectively, at a concentration of 50 µM (Figure 18).

### 2.3. Neuroprotective Activity

Neuroprotection refers to the strategies and relative mechanisms able to defend the central nervous system (CNS) against neuronal injury due to both acute (e.g., stroke or trauma) and chronic neurodegenerative disorders [58,59]. Increased oxidative stress has been recognized as a common culprit of many neurological disorders including Alzheimer’s disease, Parkinson’s disease and stroke [60,61]. The β-Amyloid (Aβ) deposition is one of the hallmarks of Alzheimer’s disease. Therefore inhibition of Aβ peptide accumulation may be a preventive strategy for Alzheimer’s disease [62].

**Figure 19:**
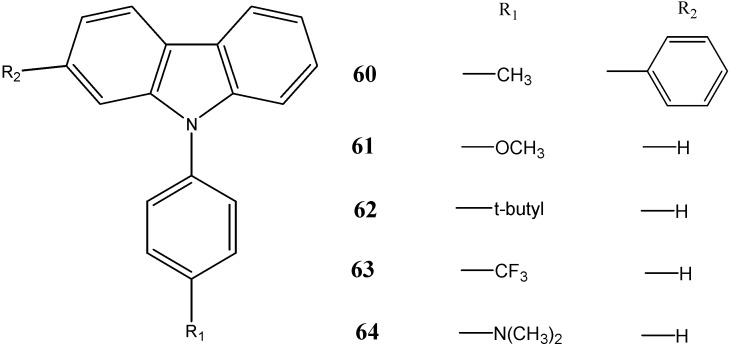
Structures of phenyl-carbazoles **60**–**64**.

Zhu *et al.* [63] in 2013 have synthesized a series of *N*-substituted carbazoles and studied their neuroprotective effects on neuronal cells HT22 against cell injury induced by glutamate or homocysteic acid. The compound 2-phenyl-9-(*p*-tolyl)-9*H*-carbazole (**60**) displayed considerable neuroprotective ability at the concentration as low as 3 µM which might result from its antioxidative activity with GSH-independent mechanism. The compounds substituted with bulky groups such as methoxy-phenyl (compound **61**), *t*-butyl-phenyl (**62**), trifluoro-phenyl (**63**), and *N*,*N*-dimethyl-phenyl (**64**) at the *N*-position of the carbazole also possessed significant neuroprotective activity at a concentration of 30 µM (Figure 19). It was observed that the presence of a substituent at the *N*-position of carbazole is essential for neuroprotective activity.

**Figure 20:**
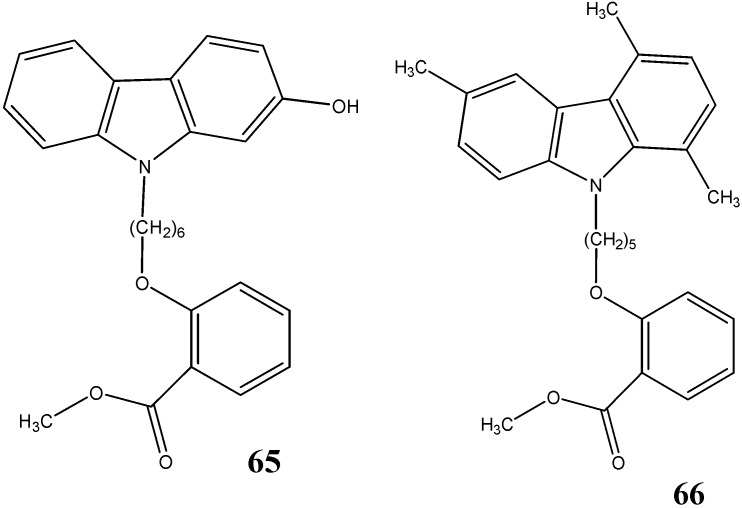
Structures of *N*-alkyl-carbazoles **65** and **66**.

A series of *N*-alkyl-carbazole derivatives synthesized by Saturnino *et al.* [64], have been investigated for their ability to promote an increase of soluble amyloid-β (Aβ) peptides. Among the tested compounds, **65** was found to be responsible for a 30%–35% increase in the concentration of soluble Aβ peptides whereas compound **66** showed 65%–70% increase in concentration of soluble Aβ peptides at 10 µM (Figure 20). In both compounds, the distance between oxygen on the chain and the *N*-atom of carbazole scaffold play a decisive role in the interaction with Aβ peptides.

**Figure 21:**
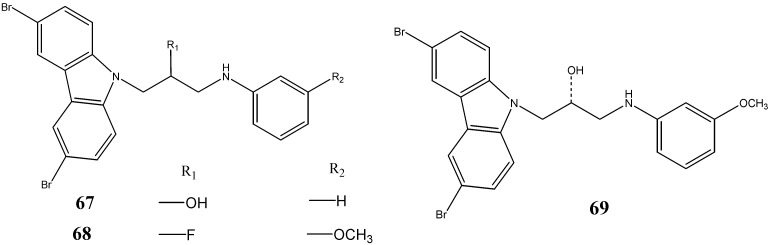
Structures of aminopropyl-carbazoles **67**–**69**.

Among many genes reported to impact adult neurogenesis is the gene encoding NPAS3, a central nervous system-specific transcription factor that is associated with learning disability and mental illness [65,66]. NPAS3 deficit mice displayed the behavioral abnormalities [67] and a profound loss of adult hippocampal neurogenesis [68]. An aminopropyl-carbazole **67**, designated as P7C3, exerts its pro-neurogenic activity by protecting newborn neurons from apoptosis and was capable of enhancing hippocampal neurogenesis in NPAS3 defecit mice. Pieper *et al.* [69] carried out a SAR study with thirty seven analogues of P7C3. The compound P7C3A20 (compound **68**) was found to possess greater potency and efficacy than P7C3, whereas the (*R*)-enantiomer of P7C3-OMe (**69**) retained an activity equivalent to that of the parent compound (Figure 21).

**Figure 22:**
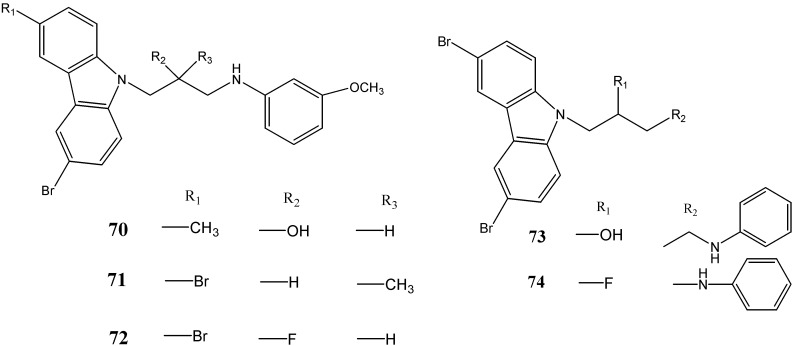
Structures of aminopropyl- and aminobutyl-carbazoles **70**–**74**.

MacMillan *et al.* [70] have developed a series of P7C3 derivatives and, among them, compounds **70**–**74** were found to be more active than the parent compound (Figure 22).

**Figure 23:**
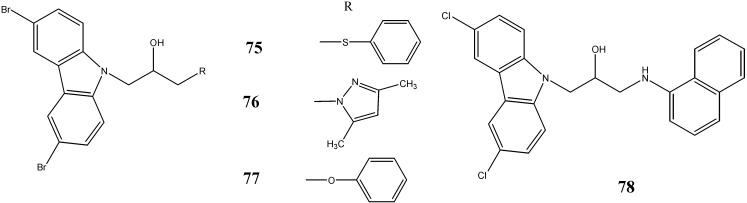
Structures of P7C3 derivatives **75**–**78**.

An aminopropyl-carbazole (P7C3) has been reported to block 1-methyl-4-phenyl-1,-2,-3,-6-tetrahydropyridine (MPTP)-mediated cell death of dopaminergic neurons in Parkinson’s disease. Cortes *et al.* [71] have screened P7C3 analogues (P7C3-S7, P7C3-S8, P7C3-S25, P7C3-S40, P7C3-S41, P7C3-S54, P7C3-S165 and P7C3-S184) for neuroprotective efficacy. The compounds P7C3-S7 (**75**), P7C3-S25 (**76**) and (*S*)-enantiomer of P7C3-S41 (**77**) were found to be active in MPTP protection assay. The compound P7C3-S184 (**78**) has been reported as a β-secretase inhibitor and prevented the formation of Aβ-peptide from amyloid precursor protein thereby proposed as a therapeutic approach for Alzheimer’s disease (Figure 23).

**Figure 24:**
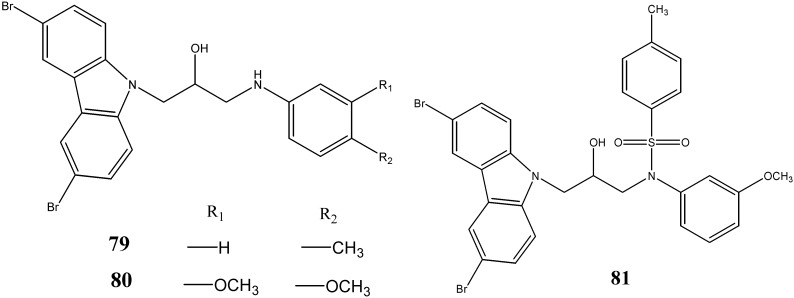
Structures of aminopropyl-carbazoles **79**–**81**.

Yoon *et al.* [72] have developed and screened 25 aminopropyl-carbazole derivatives that can enhance neurogenesis of cultured neural stem cells. Among these analogues, compounds **79**–**81** demonstrated excellent proneurogenic and neuroprotective activity with no apparent toxicity (Figure 24).

### 2.4. Anti-Epileptic and Antinociceptive Activities

Epilepsy is the most frequent neurologic infection characterized by excessive temporary neuronal discharge [73]. The overall prevalence of the disease is 1.0% of the population and up to 50 million people worldwide. Previous studies showed that a significant percent of individuals (20%–30%) using anti-epileptic drugs are resistant to the currently used therapeutic agent [74]. Therefore researchers are trying to find more active and less toxic compounds to control the seizures and produce a more comfortable life for the patients [75,76].

**Figure 25:**
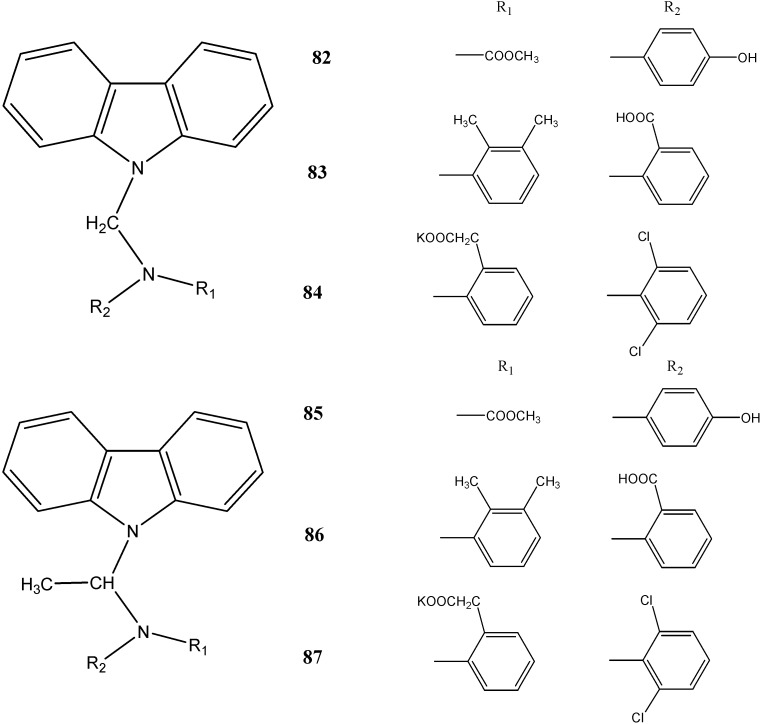
Structures of *N*-alkyl-carbazoles **82**–**87**.

The *N*-substituted carbazoles **82**–**87** have been introduced by Rjamanickam *et al.* [77] and were screened for their antilepileptic and antinociceptive activities (Figure 25). Compound **86** showed highly significant anti-epileptic potential at a concentration of 20 mg/kg. This may be due to the substituent, 2-(2,-3-dimethyl-phenyl)-amino-benzoic acid. The compounds **82**–**87** have been reported to possess analgesic potential. The details of the antinociceptive activity of these compounds are given in Table 1.

**Table 1:** Antinociceptive evaluation of compounds (**82**–**87**).

Treatment	Basel Reaction Time (s) before Treatment (Mean ± SEM)	Reaction Time (s) after Administration (Mean ± SEM)			
		15 min	30 min	60 min	120 min
Control	4.1232 ± 0.2342	4.0228 ± 0.2322	4.1244 ± 0.2478	4.3244 ± 0.2286	4.3646 ± 0.2672
Std ^a^	4.5120 ± 0.9012	8.800 ± 0.9031 **	10.22 ± 0.2642 **	11.48 ± 0.3476 **	13.22 ± 0.2974 **
**82**	4.9400 ± 0.6274	5.560 ± 0.6325 *	6.48 ± 0.2224 *	8.66 ± 0.2462 **	10.34 ± 0.2874 **
**83**	4.8819 ± 0.4654	5.042 ± 0.4761 *	6.58 ± 0.2978 *	8.44 ± 0.2536 **	10.48 ± 0.3576 **
**84**	4.7258 ± 0.4839	5.702 ± 0.4830 *	7.46 ± 0.2564 **	9.26 ± 0.2978 **	11.22 ± 0.6428 **
**85**	4.5276 ± 0.5432	5.226 ± 0.5428 **	7.62 ± 0.2464 **	8.70 ± 0.3260 **	9.86 ± 0.2642 **
**86**	4.5276 ± 0.5324	5.406 ± 0.5428 *	7.44 ± 0.4242 **	9.66 ± 0.2484 **	10.26 ± 0.2564 **
**87**	4.6863 ± 0.4912	5.863 ± 0.3726 *	7.54 ± 0.4264 **	9.02 ± 0.2478 **	10.68 ± 0.2346 **

** *p* < 0.001 *vs.* control indicates highly significant; ^a^ Pentazocine standard; * *p* < 0.01 *vs.* control indicates significant.

## 3. Conclusions

Carbazole is a unique template associated with several biological activities. Due to the diverse and versatile biological properties of carbazole derivatives, particularly *N*-substituted carbazoles, they are of great interest to the research community. In particular, their antimicrobial, anticancer and antinociceptive activities makes these compounds attractive candidates, not only for microbe borne diseases, but also for the several other conditions like Alzheimer’s disease and epilepsy. This article has presented a comprehensive review of potent *N*-substituted carbazoles reported for their biological activities. More research must be carried out to explore their therapeutic potential for many other diseases like AIDS, hepatitis and diabetes.
